# Hypervolemia for Hypertension Pathophysiology: A Population-Based Study

**DOI:** 10.1155/2014/895401

**Published:** 2014-08-11

**Authors:** Ender Hür, Melih Özişik, Cihan Ural, Gürsel Yildiz, Kemal Mağden, Sennur Budak Köse, Füruzan Köktürk, Çağatay Büyükuysal, İbrahim Yildirim, Gültekin Süleymanlar, Kenan Ateş, Soner Duman

**Affiliations:** ^1^Division of Nephrology, Bülent Ecevit University, Zonguldak, Turkey; ^2^Department of Internal Medicine, Ege University, Izmir, Turkey; ^3^Nephrology Clinic, Ataturk State Hospital, Zonguldak, Turkey; ^4^Nephrology Clinic, Istanbul Education and Research Hospital, Istanbul, Turkey; ^5^Department of Biostatistics, Bülent Ecevit University, Zonguldak, Turkey; ^6^Division of Nephrology, Akdeniz University, Antalya, Turkey; ^7^Division of Nephrology, Ankara University, Ankara, Turkey

## Abstract

*Objectives*. Hypertension and hypervolemia relationship was proven among renal disease, although it is not known in normal population. Present study determines the fluid distribution defects in relation to blood pressure. *Material and Methods*. In a population-based survey in Turkey demographics, height, weight, blood pressure, urine analysis, and serum creatinine measurements were recorded. Bioimpedance measured with the Body Composition Monitor. *Results*. Total 2034 population of 71.6% male, mean age 47 ± 12.6 (18–89) years, systolic blood pressure (SBP) 134.7 ± 20, diastolic blood pressure 77.9 ± 11.6 mmHg. Body mass index (BMI) was 28.5 ± 4.5 (15.8–50.6) kg/m^2^; overhydration was 0.05 ± 1.05 L. There was a correlation between extracellular water (ECW)/height and SBP (*r* = 0.21, *P* < 0.001). Receiver operating characteristic (ROC) curve with the performance of 0.60 (*P* < 0.001) that showed cut-off value of ECW/height was 10.06 L/m, with the 69% sensitivity and 45% specificity for SBP: 140 mmHg values. Risk factors for high SBP were increase of ECW/Height, age, BMI and presence of diabetes. ECW/height, SBP, and fat tissue index (FTI) increased in BMI categories (low, normal, and obese) and in diabetics. SBP and FTI were lower in smokers. *Conclusions*. High blood pressure may be accompanied by increased extracellular volume indices. In the future volume status assessment could be of use in evaluating the effectiveness of pharmacological intervention in the treatment of hypertension.

## 1. Introduction

Hypertension is one of the most important health problems in the world. The mortality and morbidity are related to the severity and duration; therefore, early diagnosis and treatment provide favorable clinical outcome. It has been proposed that abnormal ion transport by the kidney and subsequent disruption of body fluid volumes are responsible for the development of hypertension [1].

Etiology has been defined in only 5% of hypertensive subjects, while the essential hypertension is the classification in the majority of subjects meaning that no clear underlying cause can be found. Up to now obtaining data about body fluids have many difficulties; therefore, studies performed in this field were in small size and related to a specific disease group [2]. It is not known if these body fluid changes represent the cause or rather the effect of hypertension. Clarifying the relation of body fluid and blood pressure rise in a population study could offer new therapeutic approaches with remarkable impact on this affection.

In bioimpedance spectroscopy (BIS), complex impedance is measured over a wide range of frequencies and the data are fit to a well-known model of biological tissue. The extracellular and intracellular resistance are obtained by Cole model [3, 4]. Total body water (TBW) is the sum of ECW + ICW. The basis of BIS is that at low frequency (zero) current, there is no conduction through biological cells and only the ECW is measured and at high frequency (infinite), both the ECW and ICW are fully measured.

A new tool (FMC BCM) (based on plausible scientific principles) has been introduced to the medical field that allows the ECW and ICW (and overhydration) to be measured routinely with reasonable accuracy for the first time in history. This tool has revealed that significant overhydration (OH) is present in 30% of European HD and PD patients [5] and a strong predictor of LV hypertrophy and mortality [6, 7]. It has also been found that BP is a poor predictor of OH during heart failure [8]. The obvious next step is to study the general population with this new tool.

Aim of this epidemiologic study is to determine the assessment of body fluids and the estimation of the compartmental distribution in ICW and ECW via BIS in a healthy population and their relation to blood pressure. It is the first study to show the compartmental distribution defects with relation to blood pressure in such a large population.

## 2. Subjects and Methods

### 2.1. Study Design

We performed a population-based, national survey in Turkey on population aged over 18 years. All subjects included in this survey gave informed consent to participate in the field study. Exclusion criteria were the presence of pacemaker or defibrillator, artificial joints, pin, or amputation; presence of serious life-limiting comorbid situations, like malignancy; uncontrollable infection; end-stage cardiac, pulmonary, or hepatic disease; and pregnancy or lactating.

The study was approved by Turkish Ministry of Health and was conducted in accordance with ethical principles of the Declaration of Helsinki; all patients provided written inform consent.

### 2.2. Sampling Method

A random sampling was used to select the study participants. A sampling frame was defined as the five official geographical regions of Turkey. The study sample comprised 17 cities including both the city with the highest population and a randomly selected city with a low population in each geographical area.

### 2.3. Field Study

The data were collected through Turkish Population Renal Health Screening Programme organized by Turkish Society of Nephrology. Measurements and interviews of potential participants by specially trained field study teams (medical doctors, nurses, laboratory technicians). During interviews, the study questionnaire included questions on subject demographics current diseases and drugs, family history, and other relevant medical history. In addition, height, weight, and blood pressure were measured. Blood pressure was measured from right arm in sitting position. Bioimpedance measurements were performed in supine position by a trained medical doctor.

### 2.4. Laboratory Assessment

Spot urine analysis (by dipsticks) was performed before BCM measurement, and results were recorded on the study questionnaire. Urinary measurements were excluded in menstruating women and in all patients suffering from febrile illness [9]. Serum creatinine (alkaline picrate method) was used. Assessing proteinuria from a test strip was carried out using the Combur Test M system with an automatic reading given by a Miditron M (Roche Diagnostics) urinalysis system.

### 2.5. Measurement of Overhydration

After measurement of body weight and height and with subjects following voiding, bioimpedance spectroscopy (BIS) was measured with the Body Composition Monitor (BCM) from Fresenius Medical Care, Deutschland GmbH. Four electrodes were placed on the right hand and foot on the side contralateral to the arteriovenous fistula, of supine patients. Two electrodes were dorsally placed on the hand in the metacarpophalangeal articulations and in the corpus, respectively, 5 cm apart. The pair on the foot was located in the metatarsophalangeal and in the articulation, 6 cm apart. The BCM analyses total body electrical impedance to an alternate current (0.2 mA) with fifty different frequencies (5–1000 kHertz). First the ECW, ICW, and total body water are calculated via determining electrical resistances. Then values of OH, body mass index, lean tissue index, fat tissue index, and body cell mass are provided by the BCM software [3].

The reference ranges are defined by the 10th and 90th percentiles of the reference population and are specific to age and gender [3].

### 2.6. Definitions

Serum creatinine levels, estimated GFR, and spot urine microalbuminuria were studied as markers for kidney function. CKD was defined as kidney damage with or without a decrease in GFR, which was calculated using a simplified version of the Modification of Diet in Renal Disease (MDRD) formula [186 × (Scr) − 1.154 × (Age) − 0.203 × (0.742 if woman) × (1.212 if African American)] [10]. Since there were no African American subjects in our study population, the last variable of the formula was not used.

The fluid volumes extracellular (ECW), intracellular (ICW), and total body water (TBW) were determined using the approach described by Moissl et al. [3]. The hydration status, lean tissue mass (LTM), and fat mass were calculated based on a physiologic tissue model described by Chamney et al. [4]. LTM and Fat were normalized to the body surface area to obtain lean tissue index (LTI = LTM/height^2^) and fat tissue index (FTI = Fat/height^2^). The values for LTI and FTI were compared to an age- and gender-matched reference population (*n* = 1248) [11].

Body mass index (BMI) was calculated as weight (kg)/height (m^2^).

Hydration reference plot. By combining measurements of OH and SBP, a normal healthy population reference region (N) was established as SBP 100–140 mmHg, OH (−1.1)–(1.1) L.

Region Dx. SBP 100–150 mmHg and a typical weight gain of (−1.1)–(2.5) L. The selection of 2.5 L is completely arbitrary.

*Region I.* This region represents patients with a OH > 2.5 L and an increased SBP > 140 mmHg. There is a high likelihood that hypertension in these patients is indicative of the gross OH observed.

*Region I-II*. This represents a population with mild elevation of –OH between 1.1 and 2.5 L concomitant with an increased SBP > 150 mmHg.

*Region II*. This represents patients in a state of normohydration, but SBP > 150 mmHg. Patients in this region are clearly hypertensive, but there is far less likelihood that volume is a contributing factor.

*Region III*. It characterises underhydrated patients with normal or low SBP < 140 mmHg.

*Region IV*. This represents patients with gross OH, OH > 2.5 L, and a normal or low SBP ≤ 140 mmHg. In this patient population, the gross OH is not reflected in SBP [8].

### 2.7. Statistical Analysis

Statistical analysis was performed with SPSS 18.0 software (SPSS, Inc., Chicago, IL, USA). Continuous variables were expressed as mean ± standard deviation and categorical variables as numbers and percentages. Continuous variables were compared with the Independent Sample *t*-test or Mann-Whitney *U* test and categorical variables were compared using Pearson's Chi-square test. Pearson and Spearman correlations were used for the linear relation between two numerical dates. Receiver operating curve (ROC) analysis was used for the detection of cut-off values. Binary logistic regression analysis with forward stepwise method was used for determination of risk factors. *P* value of less than 0.05 was considered statistically significant for all tests.

## 3. Results

Study cases were aged between 18 and 89, with the mean of 47 ± 12.6 years. Characteristics of the study population were given in Table 1.

Mean BCM measurement quality given by the device itself was 93.22 ± 4.6 percent; measurement duration was 69.2 ± 33.6 seconds.

Mean systolic blood pressure (SBP) was 134.7 ± 20.0, and diastolic blood pressure was (DBP) 77.9 ± 11.6 mmHg. BMI was 28.5 ± 4.5 (15.8–50.6) kg/m^2^, body surface area (BSA) was 1.89 ± 0.17 (1.33–2.47) m^2^, OH (L) was 0.05 ± 1.05 L, OH/ECW (%) was 0.01 ± 5.94, ECW/height was 10.46 ± 1.36 L/m, TBW (L) was 39.9 ± 6.6, ECW (L) was 17.6 ± 2.79, ICW (L) was 22.3 ± 4.15, LTI (kg/m^2^) was 16.3 ± 2.85, FTI (kg/m^2^) was 11.9 ± 5.3, adipose tissue mass (ATM) was 32.82 ± 13.32 kg, lean tissue mass (LTM) was 46.44 ± 10.63 kg, and body weight was 79.2 ± 13 kg.

There was a weak positive correlation between ECW/height and SBP (*r* = 0.21, *P* < 0.001). There were a strong correlation between BMI and FTI (*r* = 0.82, *P* < 0.001), correlation even more pronounced among females (*r* = 0.93, *P* < 0.001), whereas no correlation between BMI and LTI in both genders but a weak positive correlation in male group (*r* = 0.16, *P* < 0.001). There was a negative correlation between eGFR and ECW/Height (*P* = 0.024, *r* = −0.06).

Age, BMI, ECW/height, and BSA increased significantly in normotensives than hypotensives and even increased in hypertensives than in both normotensives and hypotensives. FTI was higher in hypertensives than in both normotensives and hypotensives. Smoking rate was lower in hypertensives compared to normotensives (Table 2).

There was a statistical significance in relation to diabetes, smoking habits, and measurement time (whether morning or afternoon) in relation to SBP. In 52.5% of diabetics and 25.8% of cigarette smokers, 32.1% of afternoon measurements had SBP ≥ 140 mmHg, and in 68.2% of nondiabetics and 64.9% of nonsmokers, 61.2% of morning measurements had SBP < 140 mmHg (*P* < 0.05).

**Figure 1:**
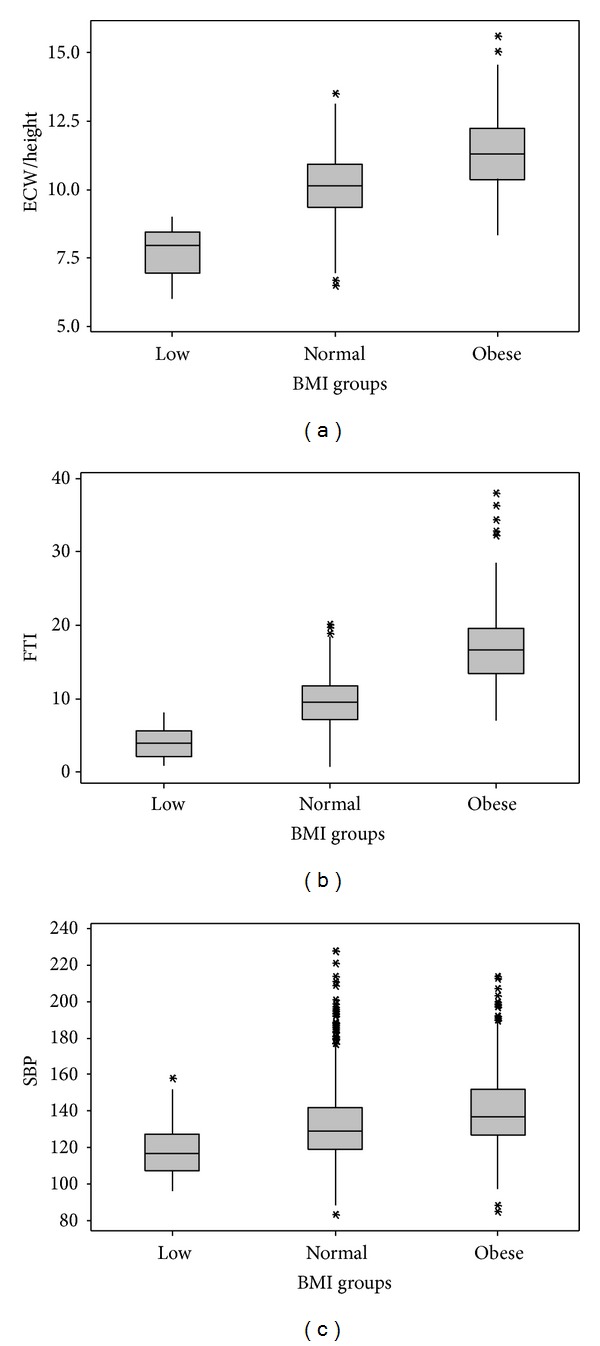
Obesity related with hydration fat tissue index and systolic blood pressure. *P* < 0.001 between all groups. ECW: extracellular water/height (L/m^2^), FTI: fat tissue index (kg/m^2^), SBP: systolic blood pressure (mmHg), and BMI: body mass index (kg/m^2^).

**Figure 2:**
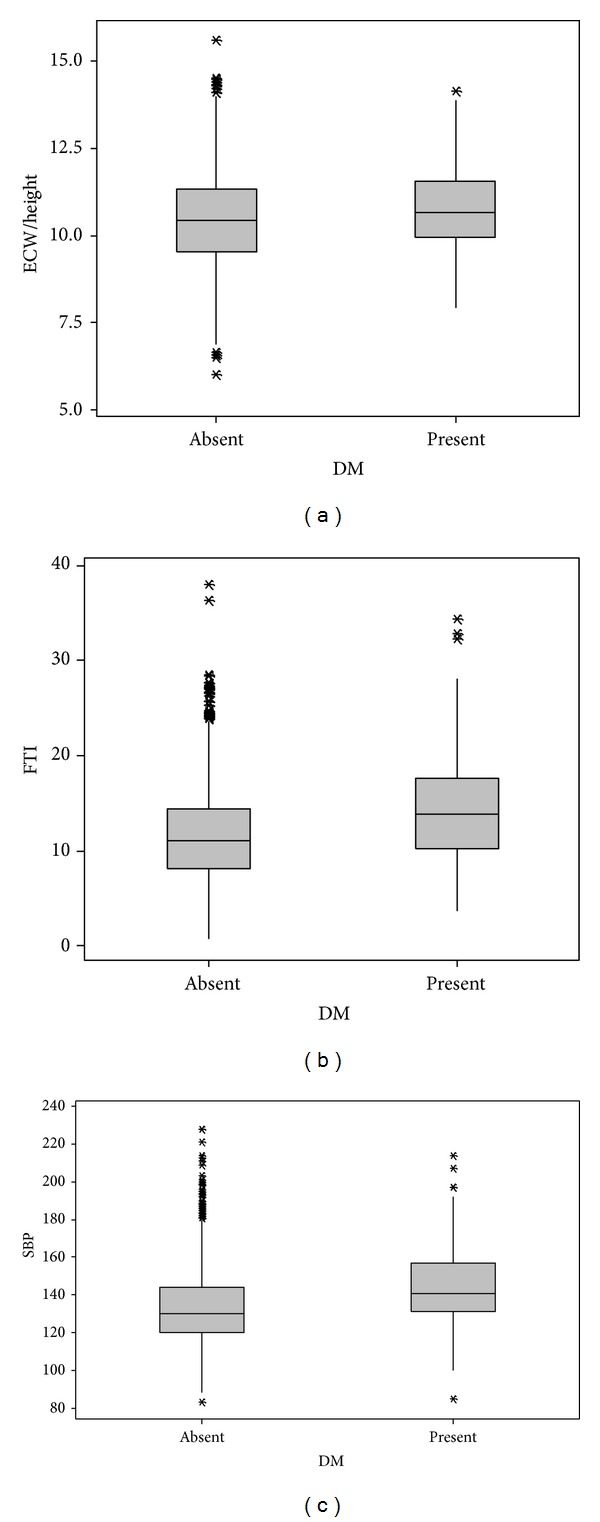
Diabetes mellitus related to hydration fat tissue index and systolic blood pressure. *P* < 0.001 between all groups. ECW: extracellular water/height (L/m^2^), FTI: fat tissue index (kg/m^2^), SBP: systolic blood pressure (mmHg), and BMI: body mass index (kg/m^2^).

**Figure 3:**
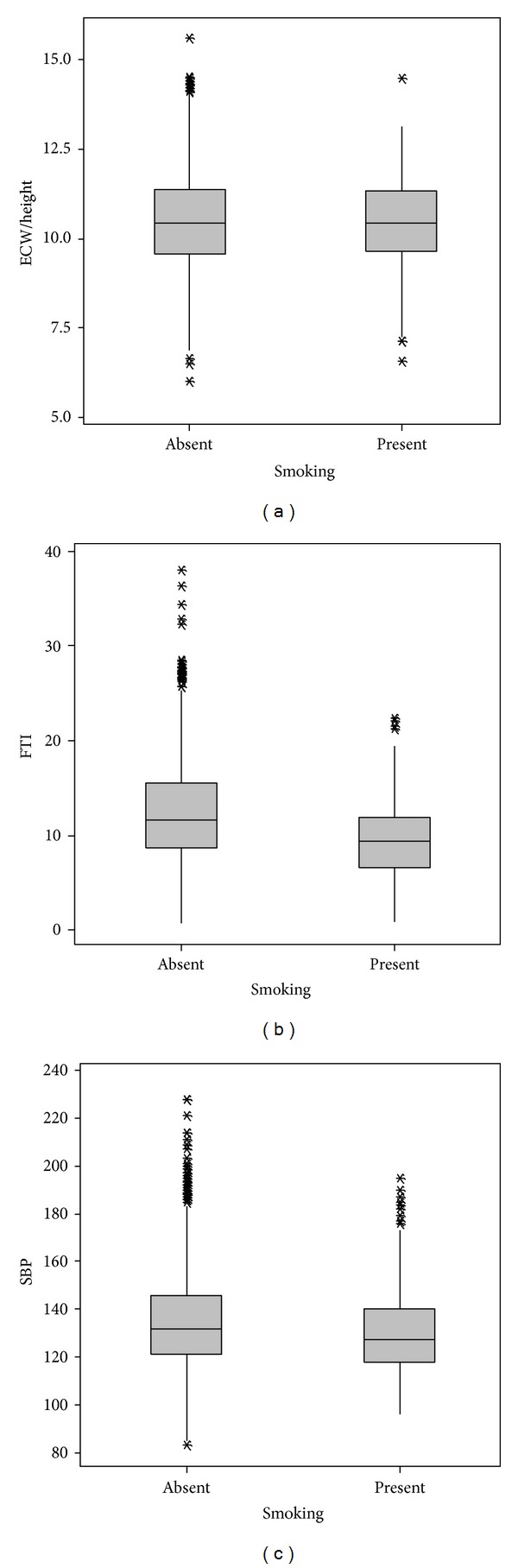
Smoking related to hydration fat tissue index and systolic blood pressure. *P* < 0.001 for FTI and SBP. ECW: extracellular water/height (L/m^2^), FTI: fat tissue index (kg/m^2^), SBP: systolic blood pressure (mmHg), and BMI: body mass index (kg/m^2^).

Risk factors for high SBP were increase of ECW/height, age, and BMI and presence of diabetes (Table 3). ECW/height 7.71 ± 0.87, 10.1 ± 1.17, 11.32 ± 1.28 L/m, FTI 4 ± 2.2, 9.5 ± 3.5, 17.1 ± 4.7 kg/m^2^ and SBP 120 ± 17, 132 ± 19, 140 ± 21 mmHg, increased in as BMI increased (low, normal and obese), respectively, (*P* < 0.001) (Figure 1). ECW/height of 10.81 ± 1.24 and 10.41 ± 1.37 L/m, FTI of 14.7 ± 5.8 and 11.5 ± 5.1 kg/m^2^, and SBP of 144 ± 22 and 133 ± 19 mmHg increased in diabetics compared to nondiabetics (*P* < 0.001) (Figure 2). ECW/height of 10.44 ± 1.29 and 10.47 ± 1.37 L/m (*P* > 0.05), FTI of 9.5 ± 4.1  12.3 ± 5.4 kg/m^2^ (*P* < 0.001), SBP of 131 ± 18, 9.5 ± 4.1, and 135 ± 20 mmHg (*P* < 0.001) were lower in smokers than in nonsmokers (Figure 3).

Mean overhydration (OH) measured in the afternoon was more than morning measurements (0.0957 ± 1.03963 versus −0.0584 ± 1.06571, (*P* = 0.001)).

Receiver operating characteristic (ROC) curve with the performance of 0.60 (*P* < 0.001) that showed cut-off value of ECW/height was 10.06 L/m, with the 69% sensitivity and 45% specificity for SBP: 140 mmHg values.

**Figure 4:**
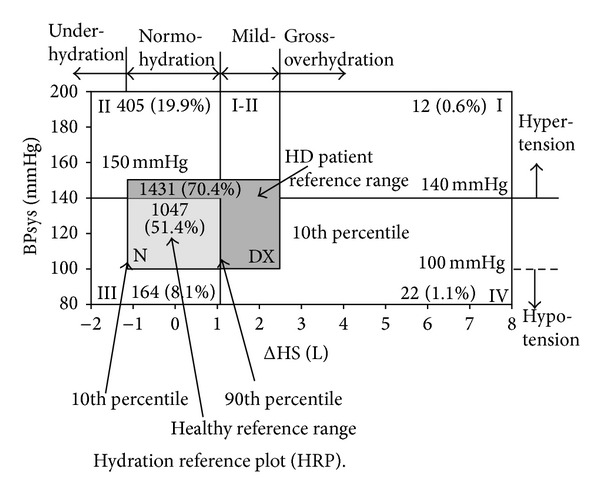
Hydration reference plot.

According to hydration reference plot, there were 12 (0.6%) cases in Region 1, 405 (19.9%) in Region 2, 164 (8.1%) in Region 3, 22 (1.1%) in Region 4, 1431 (51.4%) in DX, and 1047 (51.4%) in Normal region (Figure 4). All cases were compared with DX region; in Region 1 there were no cases with CAD and increased incidence of DM 33.3% (*P* < 0.05). In Region 2 increased incidence of DM and CAD and increased BMI (20.3%, 7.7% and 29.7 kg/m^2^, resp.) (*P* < 0.001). In Region 3 increased BMI 28.6 kg/m^2^(*P* < 0.001). In Region 4 increased incidence of CAD 13.6%, (*P* < 0.001). (Table 4).

## 4. Discussion

Hypertension is a public health problem. According to recent meta-analysis including 966 patients with masked hypertension (MH) and 2640 healthy controls with sustained normotension, controlled hypertension, and white coat hypertension, the prevalence of LVH ranged from 7 to 66% in MH and from 0.4 to 42% in non-MH counterparts (average 29 versus 9%, *P* < 0.01) [12]. Definition of the special risk factors (genetics, dietary habits, and life style), leading causes, and early diagnosis are important for prevention of the disease for individual population.

Bioelectrical impedance spectroscopy is a noninvasive, inexpensive, and portable method that has been used mainly for body-composition analysis over the past decade. It is a suitable tool for large epidemiological studies. Our data showed that high blood pressure subjects had increased volume parameters which are consistent with previous observations [13–16].

In present study E/I was higher in hypertensive group. But we know that E/I could be affected by changes in ICW as well as by ECW varying with hydration. This may lead to the spurious impression of overhydration in subjects with smaller ICW volumes so it does not reflect hydration alone [17]. Therefore we used other methods of expressing ECW as a measure of hydration.

Bomback et al. has reported that kidney handicap results in an altered aldosterone-ECV relationship that extends into the general population having negative volume, load, and inflammatory effects [18–20]. The expanded ECW in haemodialysis patients and the markedly elevated aldosterone levels seen in ESRD [18]. Low-dose mineralocorticoid receptor blocker therapy which provided better control of subclinical ECW expansion may attenuate the adverse effects of this receptor activation [19]. In obesity aldosterone level elevation and extracellular volume expansion are crucial for renal disease via aldosterone's nonepithelial, profibrotic, and proinflammatory effects [20]. In present study BMI highly correlated with FTI measured by bioimpedance. Obese people have significantly higher SBP than normal and low BMI group. Tagliabue et al. investigated the differences in the relationship between multifrequency impedance and body-water compartments (total body water (TBW) and extracellular water (ECW)) measured by dilution techniques in Italian and Dutch healthy subjects aged 19–41 years. In body build between the two groups, the main differences were height, trunk length, and the two ratios TBW/height and ECW/height. Population-specific prediction formulas for ECW (at 1 kHz) and TBW (at 100 kHz) were developed. The prediction errors for ECW and TBW were about 0.6 and 1.5 kg, respectively, in both groups. They concluded that the water distribution between the extra- and intracellular compartments was the major cause of error in the prediction of body water, and in particular of ECW from impedance measurements with a population-specific equation [21]. Deurenberg et al. found that different, more slender body build leads to overestimation of ECW of the Ethiopian population. That study indicated that the validity of predicted body water from impedance depends on the body build of the subjects, which should be taken into account to avoid systematic errors when applying prediction formulas from a reference population to another population under study [22]. Bartz et al. showed ECW measured by multifrequency impedance was underestimated in males and slightly overestimated in females of Indonesian people and concluded that the validation in a larger group of related population subjects was needed [23]. But at the same time it has been discovered that OH, as determined by ECW/Ht, is highly predictive of clinical disease. ECW/height correlated well with volume overload as assessed by echocardiography in PD patients [24, 25]. In the present study there was no obvious relationship between OH and blood pressure, but this might be expected because OH is calculated from the difference between the measured ECW and that expected which refer to patients of same age and sex, but they do not take into account racial differences.

A decrease in vascular compliance, such as what occurs with aging in larger blood vessels such as the aorta, may also contribute to the development of isolated systolic hypertension. Even in these conditions, however, it is quite possible that the kidney plays a role in maintaining the hypertensive response [26]. In present study we also found that advanced age and diabetic groups have high blood pressure.

Visser et al. showed that young healthy men with a higher BMI are associated with a larger increase in ECW during high salt intake, suggesting that altered sodium and fluid handling may be an early phenomenon in the pathophysiological consequences of weight excess and that dietary sodium restriction may have preventive potential in overweight subjects [27]. In present study BMI and FTI were higher in high blood pressure group. For every 10% increase in body fat, OH decreased by 1.2 L; obesity seems to afford some protection aginst OH in dialysis patients [28].

In present study high blood pressure group have lower GFR measured by MDRD. In the literature the relation of ECW increase and a positive correlation between ECW and HT in patients with poor renal function were demonstrated [29, 30]. Recently we also demonstrated the strong positive correlation of increased ECW and hypertension in peritoneal dialysis patients [25].

Diabetes history, smoking, and bioimpedance measurement time afternoon were all associated with increased ECW. Brizzolara et al. demonstrated that poor glucose control positively correlated with ECW and E/I ratio [31]. These observations proving that good or moderate long-term control IDDM patients have proportionately normal distributions of ECW and ICW excess. Osmotic effect of glucose may be responsible.

A causal relationship between exposure to smoking and increase in blood pressure (BP) is not yet clearly demonstrated [32]. In a study 2742 Turkish adults prospectively evaluated for more than 7 years revealed that current cigarette smoking played a protective role at borderline significance but former smokers uniformly exhibited significantly higher risk for the development of hypertension. In our study we took only currently smoking cases as smokers, so lower blood pressure in smokers may be due to current smokers [33]. Fat tissue index was also found less than expected in smokers.

In present study for the cut-off value 10.06 L/m of ECW/height; high blood pressure predicted positively in 69% and excluded in 45%.

To our knowledge, there were no such studies comparing the effect of time period on bioimpedance measurement. We revealed that afternoon measurements were associated with increased OH, may be due to circadian rhythm of cortisone or likely whole body measurements [34]. The other reason may be the fluid shifts to the extremities during standing. It is better to tailor bioimpedance measurement protocols accordingly after such epidemiological studies.

Although the cross-sectional design of our study and other limitations preclude to infer a causal relationship between high blood pressure and ECW/height ratio, increased age and BMI and diabetes history. All these risk factors must be taken into consideration for hypertension.

There is no satisfying explanation of changes of body water composition in high blood pressure. BIS a noninvasive, cheap, and easily repeatable method has the potential to improve the etiology and management of the various hypertensive states in the majority of patients all over the world.

Bioelectrical impedance analysis is a practical method for the followup of antihypertensive therapy in pregnancy [35] and in the future it might be used for all hypertensive subjects evaluating the effectiveness of pharmacological treatments by means of volume restoration accordingly.

Expected OH in the BCM device used is setup from a largely Caucasian population, but it remains to be demonstrated whether there are differences in hydration status in different ethnic groups. In the Turkish population however the results indicate no bias in hydration when measured with BCM. So other population studies will be needed in this field.

More than half of the studied population is in “N” region of the hydration reference (Wabel plot). Most of the cases are in DX region (includes N region) 70.4%. Region 1 (overhydrated-hypertensives) composed of a minor group of population (0.6%) and having statistically significant DM and absence of CAD. Hypertension may be volume dependent just as seen in most dialysis patients. In this group hypertension treatment strategy must be adjusted towards diuretics. Region 2 (normo/hypovolemic-hypertensives) composed of nearly 1/5 of the population and having statistically significant DM, CAD and obese cases in this group. Etiology of hypertension seems as volume independent and may be speculated as rennin dependent. Accordingly angiotensin converting enzyme inhibitors and angiotensin receptor blockers might be the first choice for probable etiology and related comorbidities. Region 3 (underhydrated-normal/low SBP) composed of 8.1% of whole population with increased number of obesity. Region 4 (overhydrated-normo/hypotensives) composed of 1.1% of population with increase incidence of CAD. This group needs further cardiac evaluation for congestive heart diseases and salt restriction must be logical approach for such cases.

Normal BP in the presence of overhydration would also be an important group to identify/screen to target treatment strategies appropriately. Hypertensive subjects who are not overhydrated would likely need different treatment strategies also. Measuring BP and hydration and taking into account the traditional risk factors may allow for better diagnosis and treatment. Blood pressure and hydration can be measured readily and this could help to direct the line of investigation in the search for primary causes.

High blood pressure may be accompanied by increased extracellular volume indices. In the future volume status assessment could be of use in evaluating the effectiveness of pharmacological intervention in the treatment of hypertension.

## Figures and Tables

**Table 1:** Characteristics of the study population (*n* = 2034).

	*n* (%)
Gender	
Male	1456 (71.6)
Female	578 (28.4)
Age groups	
<65	1870 (91.9)
≥65	164 (8.1)
Geographical region	
Central Anatolia	323 (15.9)
Mediterranean	141 (6.9)
Aegean	578 (28.4)
East Anatolia	626 (30.8)
Southeastern Anatolia	366 (18)
Measurement time	
Morning (<12 o'clock)	649 (31.9)
Afternoon (≥12 o'clock)	1385 (68.1)
Disease history	
Healthy	1250 (61.5)
Diabetics	240 (11.8)
Known HT	380 (18.7)
CAD	87 (4.3)
COPD	41 (2)
CKD	47 (2.9)
Anti-HT	
ACEi/ARB	96 (4.7)
Beta blockers	50 (2.5)
CCB	18 (0.9)
Diuretics	7 (0.3)

The number of subjects is crude (unadjusted) figures.

HT: hypertension, CAD: coronary artery disease, COPD: chronic obstructive pulmonary disease, CKD: chronic kidney disease, ACEi: angiotensin converting enzyme inhibitors, ARB: angiotensin receptor blockers, and CCB: calcium channel blockers.

**Table 2:** Univariate analysis of systolic blood pressure categories.

Variables	Hypotensives (SBP < 110 mmHg) (*n* = 133)	Normotensives (140 > SBP ≥ 110 mmHg) (*n* = 1208)	Hypertensives (SBP ≥ 140 mmHg) (*n* = 693)
BMI (kg/m²)	26.38 ± 4.98	27.59 ± 4.39 a	29.49 ± 4.23 a∗ b∗
ECW/height (L/m)	9.93 ± 1.58	10.34 ± 1.32 a∗	10.78 ± 1.32 a∗ b∗
Age	41.47 ± 11.69	44.36 ± 12.38 a	52.84 ± 11.23 a∗ b∗
DM	0.06 ± 0.24	0.09 ± 0.28	0.18 ± 0.39 a∗ b∗
Smoking	0.16 ± 0.37	0.16 ± 0.37	0.11 ± 0.31 b
FTI (kg/m²)	10.46 ± 5.12	11.22 ± 5.26	13.23 ± 5.14 a∗ b∗
BSA (m²)	1.82 ± 0.19	1.88 ± 0.17 a∗	1.91 ± 0.16 a∗ b∗
TBW (L)	38.10 ± 7.14	39.88 ± 6.56 a	40.29 ± 6.64 a∗
OH (L)	0.09 ± 1.09	0.03 ± 1.01	0.07 ± 1.11

*P* < 0.05; a: group versus hypotensives; b: group versus normotensives; *P < 0.001.

SBP: systolic blood pressure, BMI: body mass index, ECW: extracellular water, DM: diabetes mellitus, FTI: fat tissue index, BSA: body surface area, TBW: total body water, and OH: overhydration.

**Table 3:** Logistic regression analysis for “high systolic blood pressure.”

Variables	Odds ratio	95% CI	*P* value
DM	1.581	1.177–2.124	0.002
ECW/height	1.104	1.003–1.215	0.043
BMI	1.058	1.027–1.090	0.000
Age	1.053	1.043–1.063	0.000

DM: diabetes mellitus, ECW: extracellular water, BMI: body mass index, and CI: confidence interval. In model: OH/ECW, E/I, TBW, ECW/height, age, gender, BMI, LTI, FTI, DM, time, and smoking.

**Table 4:** Comorbidities according to hydration reference plot.

Hydration reference plot regions	BMI (median)	DM (%)	CAD (%)	CKD (%)
1 (*n* = 12)	30 (24.8–36.5)	33.3 a	0 a∗	9.1
2 (*n* = 405)	29.7 (18.1–41.6) a∗	20.3 a∗	7.7 a∗	4.6
3 (*n* = 164)	28.6 (18.3–50.6) a∗	6.71	2.4	0
4 (*n* = 22)	26.9 (17.5–36.9)	18.2	13.6 a∗	0
DX (*n* = 1431)	27.5 (15.8–45.5)	9.7	3.4	2.7

Total (2034)	28 (15.8–50.6)	11.8	4.3	2.9

*P* < 0.05; a: group versus DX. *P < 0.001.

BMI: body mass index, CAD: coronary artery disease, CKD: chronic kidney disease, and DX: overhydration between −1.1 and 2.5 L with systolic blood pressure 100–150 mmHg.
